# Genetically Confirmed Familial Case of Nonsyndromic Cardiac Progeria Caused by the *LMNA* p.Asp300Asn Variant with Presumed Gonadal Mosaicism: Phenotypic Comparison with Previously Reported Patients

**DOI:** 10.3390/genes16111250

**Published:** 2025-10-22

**Authors:** Ekaterina Nuzhnaya, Margarita Sharova, Uliana Chubykina, Anna Orlova, Ekaterina Vorontsova, Peter Vasiliev

**Affiliations:** 1 Federal State Budgetary Institution, Research Centre for Medical Genetics, Moskvorechye Str. 1, Moscow 115522, Russiavasiluev@med-gen.ru (P.V.); 2 National Medical Research Center of Cardiology Named After Academician E.I. Chazov, Ministry of Health of the Russian Federation, Academician Chazov Str. 15a, Moscow 121552, Russia

**Keywords:** *LMNA*, laminopathy, nonsyndromic cardiac progeria, gonadal mosaicism

## Abstract

We describe the first genetically confirmed familial case of nonsyndromic cardiac progeria caused by the *LMNA* NM_170707.4:c.898G>A (p.Asp300Asn) variant, with evidence suggesting gonadal mosaicism as the mechanism of inheritance. The proband developed severe early-onset valvular and coronary artery disease requiring multiple surgical interventions, yet showed no systemic progeroid features. Genetic analysis revealed the heterozygous variant in her unaffected daughters and nieces but not in either parent. Comparison with previously reported cases highlights striking clinical heterogeneity associated with this variant, ranging from isolated cardiovascular involvement to syndromic progeroid manifestations with metabolic and skeletal abnormalities. This variability likely reflects the combined influence of genetic, epigenetic, and environmental factors. Our case expands the clinical spectrum of *LMNA* p.Asp300Asn and underscores the importance of considering laminopathies in patients with unexplained early-onset cardiac disease. Early genetic diagnosis is essential for the management, surveillance, and counseling of affected families.

## 1. Introduction

The *LMNA* gene encodes nuclear lamins A and C, key structural proteins of the nuclear lamina involved in maintaining nuclear architecture, chromatin organization, and the regulation of gene expression [1,2]. Pathogenic variants in *LMNA* are associated with a wide spectrum of disorders collectively referred to as laminopathies, encompassing cardiomyopathies, muscular dystrophies, lipodystrophies, neuropathies, and premature aging syndromes such as Hutchinson–Gilford progeria [3,4,5,6,7,8].

The heterozygous *LMNA* NM_170707.4:c.898G>A (p.Asp300Asn) variant has been previously reported in several independent cases with markedly variable clinical phenotypes [4,9,10,11]. While some patients present with isolated cardiac disease—such as valvular defects, coronary artery disease, or arrhythmias—defined as nonsyndromic cardiac progeria, others exhibit systemic features of progeroid syndromes, including characteristic facial dysmorphism, lipodystrophy, sclerodermoid skin changes, and metabolic disturbances such as diabetes or hyperlipidemia.

In this study, we present the first reported familial case of nonsyndromic cardiac progeria caused by the *LMNA* p.Asp300Asn variant with presumed gonadal mosaicism. In addition, we performed a detailed comparison of the clinical features observed in our case with those reported in previously published patients carrying the same variant. Our findings confirm the pronounced clinical heterogeneity from isolated cardiovascular associated with *LMNA* p.Asp300Asn, ranging involvement to systemic progeroid manifestations.

## 2. Materials and Methods

1. Clinical data: the proband was examined at the Research and Counseling Department of the Research Centre for Medical Genetics (RCMG, Moscow) at the National Medical Research Center of Cardiology named after Academician E.I. Chazov (NMRC of Cardiology, Moscow). Initial evaluation included comprehensive medical history, detailed physical examination. The diagnostic work-up comprised blood tests, instrumental investigations and interventional procedures.

Genetic analyses included whole-exom sequencing (WES). For validation and confirmation of the presence ofvariant, Sanger sequencing method with forward and reverse primers was applied. The sequencing was conducted using the protocol of the manufacturer on the ABI Prism 3100 instrument (Applied Biosystems (Thermo Fisher Scientific, Waltham, MA, USA)).

2. Genetic testing: Blood samples from the proband and unaffected parents, daughters and nieces were collected, and genomic DNA was extracted by standard methods.

DNA analysis of the patient was performed using the DNBSEQ-T7 genetic analyzer with paired-end 150 bp reads (PE150). Library preparation was carried out using a PCR-free protocol with enzymatic fragmentation (MGI). Variant nomenclature followed the recommendations provided by the Human Genome Variation Society (HGVS) as presented at http://varnomen.hgvs.org/recommendations/DNA, version 20.05.

Sequencing data processing was performed using the “NGS-data-Genome” software developed by the Bioinformatics Department of the Academician N.P. Bochkov Medical Genetic Research Center (state registration number № 2021662113).

To assess the population frequencies of the identified variants, data from the 1000 Genomes Project and the Genome Aggregation Database (gnomAD v4.1.0) were used. Clinical relevance of the detected variants was evaluated using the OMIM database, the Human Gene Mutation Database (HGMD^®^ Professional, version 2022.1), disease-specific databases (when available), and published literature.

The coding sequence of *LMNA* was completely covered when using this method. The sequencing data processing was performed using the standard automated algorithm offered by the NGSdata software v.2022.1 developed by N.S. Beskorovainy. The NGSdata software was registered under the number 2021614055 in 2021.

Aligned sequences were visualized with Integrative Genomics Viewer (IGV) browser. Filtering of the variants was based on their frequency of less than 1% in gnomAD and coding region sequence effects such as missense, nonsense, coding indels, and splice sites. The variant’s clinical significance was evaluated according to the ACMG/AMP criteria. The revealed variant was named according to the reference transcript variant (NM_170707.4) for the *LMNA* gene. Sanger sequencing was carried out to validate the variant in the proband and to assess its presence in the proband’s parents, daughters, and nieces. To amplify the fragment encompassing the candidate variant, custom primers were used:

*LMNA_5F*:CTATGCCTTCTGGGGATCAGGC

*LMNA_5R*:ACTCCACATCCTGCGACCCT

For paternity testing, a molecular genetic analysis was performed using a reagent system designed for the multiplex PCR amplification of ten short tandem repeat (STR) loci and the amelogenin gene from human genomic DNA. The analyzed STR loci included D3S1358, TH01, D12S391, D2S441, D7S820, D13S317, TPOX, D18S51, and VWA.

A literature review was performed following PRISMA guidelines in a simplified form. The PubMed database was systematically searched using the keywords “*LMNA*” and “*LMNA* p.Asp300Asn” covering all years available up to December 2024. Titles and abstracts were screened to identify reports describing human patients with pathogenic or likely pathogenic variants in *LMNA*. Duplicates, review articles, experimental or animal studies, and papers lacking clinical or genetic data were excluded.

## 3. Case Presentation

The proband, a 41-year-old woman, was referred to a clinical geneticist because of valvular and coronary artery disease since a young age. She was born to non-consanguineous parents with a positive family history. Her brother died of pulmonary embolism at the age of 32, and her sister had a history of coronary artery disease (CAD) and died at the age of 43. Unfortunately, no additional clinical data, phenotypic description, or detailed diagnostic information were available for either sibling. Her father was diagnosed with CAD and underwent coronary artery bypass grafting (CABG) at the age of 63. Notably, he had a long history of smoking and arterial hypertension, whereas the siblings did not have any conventional risk factors for cardiovascular disease.

At the age of 26, the proband presented with aortic valve stenosis and mitral valve insufficiency during her third pregnancy. At the age of 31, she was diagnosed with hypertension, with peak blood pressure readings reaching 190/100 mmHg. At the age of 36, she underwent surgical intervention for aortic and mitral valve replacement. Since the age of 39, she has presented with clinical signs of angina. Coronary angiography revealed significant stenotic atherosclerosis: 80% in the left anterior descending artery, 50% in the circumflex branch, 90% in the ostium of the right coronary artery, and 80% in its mid-segment. She was also diagnosed with atherosclerotic peripheral artery disease, with stenosis of 35% in the left internal carotid artery and 40–45% in the right common femoral artery. Due to persistent symptoms of coronary artery disease, she subsequently underwent CABG. She did not exhibit any conventional risk factors for atherosclerosis or arterial hypertension, such as dyslipidemia, smoking, diabetes mellitus, or obesity.

Physical examination revealed no signs of a systemic progeroid phenotype. A comprehensive clinical and laboratory evaluation likewise showed no evidence of lipodystrophy or neuromuscular involvement. The proband did not present with any additional abnormalities such as hepatic steatosis, insulin resistance, hypertriglyceridemia, or recurrent pancreatitis, and had a normal body weight with no metabolic risk factors. Furthermore, no evidence of osteoporosis was detected. Given the early onset of atherosclerotic disease in the absence of conventional cardiovascular risk factors, the patient was referred for genetic evaluation to investigate a potential hereditary etiology.

Molecular genetic testing of the proband was performed using WES, which identified a heterozygous variant in the *LMNA* gene, NM_170707.4:c.898G>A, located in exon 5 and predicted to be deleterious. This variant was absent from the reference alleles of the Genome Aggregation Database (gnomAD v4.0.0). The variant results in a missense substitution, p.Asp300Asn, affecting a highly conserved residue within the coil 2B subdomain of the α-helical rod domain—a region essential for head-to-tail dimerization and maintenance of nuclear lamina integrity. According to the ACMG/AMP guidelines [12], the NM_170707.4:c.898G>A variant meets criteria PM2, PP3, PM1, and PP4 and is classified as likely pathogenic, considered the cause of disease in this individual.

**Figure 1:**
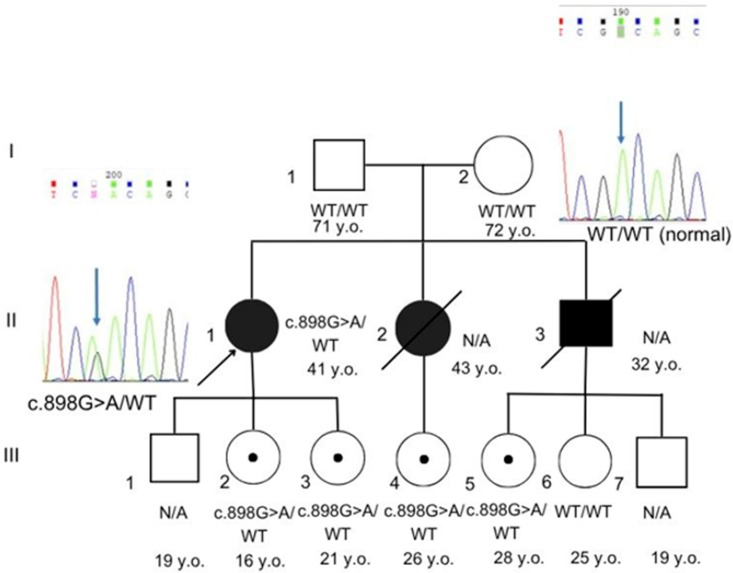
Pedigree and Sanger sequencing. Sanger chromatograms showing the heterozygous NM_170707.4 (*LMNA*):c.898G>A in the proband and asymptomatic carrier relatives (two daughters and two nieces). The variant is absent in both parents, who are wild type (WT) at this locus. Individuals II-2 and II-3, III-1, III-6, and III-7 were unavailable for further investigation. N/A not available.

For validation and segregation analysis, Sanger sequencing was performed in the proband and her family members, including her parents, daughters, and nieces. Sanger sequencing confirmed the variant as de novo with verified paternity. The heterozygous state of this variant was also detected in two unaffected daughters of the proband and two unaffected nieces, as shown in Figure 1.

The presence of the *LMNA* variant in the proband, her daughters, and nieces—who are still young and have not yet reached the typical age of clinical manifestation—suggests that the deceased siblings, whose DNA was not available for testing, may also have carried the same variant. This observation raises the possibility that the early cardiovascular deaths of the proband’s brother and sister could be associated with a germline variant in the *LMNA* gene. The absence of this variant in the peripheral blood of both parents suggests that gonadal mosaicism could represent a plausible mechanism of inheritance in this family. However, since DNA testing of buccal epithelial cells from the parents and a sperm sample from the father could not be performed, the possibility of mosaicism cannot be conclusively confirmed. Detailed clinical and laboratory data of our proband and previously reported patients are presented in Table 1.

**Figure 2:**
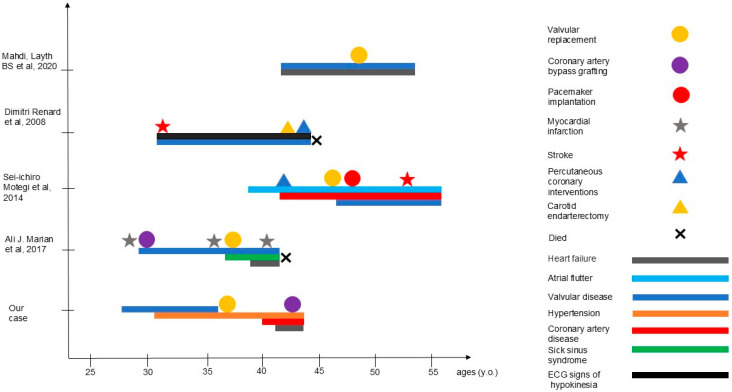
Timeline of disease onset, surgical interventions, and outcomes in the proband and previously reported cases.

The age of disease onset, clinical course, and surgical interventions in our proband, as well as in previously reported patients, are summarized in Figure 2.

Currently, the proband is 43 years old and presents with clinical signs of heart failure. Her daughters and nieces have been advised to undergo comprehensive cardiological evaluation and regular follow-up to enable the early detection of potential disease manifestations.

## 4. Discussion

Lamins are the principal structural proteins of the nuclear lamina, forming a filamentous network beneath the inner nuclear membrane that maintains nuclear stability and regulates chromatin organization, transcription, and DNA repair. Lamins A and C, encoded by the *LMNA* gene, share a central α-helical rod domain that mediates dimerization through coiled-coil interactions, forming the basic unit of higher-order filament assembly. Disruption of this structure compromises nuclear integrity, particularly in mechanically active tissues such as cardiac and vascular muscle, leading to cell fragility and apoptosis. Such mechanisms underlie the pathogenesis of a broad spectrum of laminopathies, including cardiomyopathy, muscular dystrophy, and progeroid syndromes [13].

The *LMNA* NM_170707.4:c.898G>A (p.Asp300Asn) variant identified in our proband affects the highly conserved coil 2B subdomain within the α-helical rod domain—an essential region for head-to-tail dimerization and maintenance of the nuclear lamina. Substitution of a negatively charged aspartic acid with a neutral asparagine is predicted to disrupt critical electrostatic interactions required for proper filament polymerization, resulting in nuclear envelope instability. Impaired lamin assembly increases nuclear deformability and induces DNA damage responses, chromatin disorganization, and transcriptional dysregulation [14]. These cellular effects ultimately lead to premature senescence and apoptosis, producing cardiomyopathic and progeroid phenotypes characteristic of *LMNA*-associated disorders. Similar mechanisms are well-documented in Hutchinson–Gilford progeria syndrome (HGPS), in which the *LMNA* c.1824C>T (p.G608G) variant causes aberrant splicing and accumulation of the toxic lamin A isoform, progerin, leading to progressive vascular calcification and atherosclerosis [15].

As summarized in Table 1, all previously reported patients carrying the same *LMNA* NM_170707.4:c.898G>A (p.Asp300Asn) variant are included, encompassing those with predominant cardiovascular involvement—such as early-onset valvular disease, coronary artery disease, and hypertension—as well as individuals exhibiting additional systemic or progeroid manifestations. These include metabolic abnormalities (lipodystrophy, hepatic steatosis, insulin resistance, hypertriglyceridemia, and recurrent pancreatitis) and skeletal features such as early-onset osteoporosis, reflecting the variable expressivity of this variant [4,9,10,11]. In contrast, our proband exhibited an isolated cardiac phenotype, supporting the concept of nonsyndromic cardiac progeria and highlighting the phenotypic heterogeneity associated with the *LMNA* p.Asp300Asn substitution.

Additional variants affecting other subdomains of the α-helical rod region, such as *LMNA* c.412G>C (p.Glu138Gln) within coil 1B, have also been linked to atypical progeria primarily manifesting as premature valvular heart disease [16]. These observations emphasize the functional significance of the α-helical rod domain as a determinant of cardiac and vascular integrity, where defective lamin dimerization triggers nuclear instability, mechanical failure, and apoptosis in tissues subjected to repetitive stress. In numerous cases reported by Doubaj et al., various *LMNA* variants resulting in distinct amino acid substitutions were associated with atypical progeroid phenotypes [17]. However, isolated cardiac involvement—such as valvular pathology or diffuse atherosclerosis—was not observed in those patients. Instead, the reported cases predominantly exhibited multisystem manifestations, including lipodystrophy, hepatic steatosis, insulin resistance, and osteoporosis, underscoring the broad phenotypic heterogeneity characteristic of *LMNA*-related progeroid disorders.

Although the proband initially appeared to represent a sporadic case, identification of the same heterozygous variant in her daughters and nieces—together with confirmed paternity and the absence of the variant in both parents’ blood DNA—suggests that gonadal mosaicism in one of the parents could represent a plausible mechanism of inheritance. However, as additional tissues such as sperm or buccal epithelium were unavailable for analysis, this possibility cannot be definitively confirmed. Notably, although the proband’s father had coronary artery disease requiring bypass surgery, this was likely attributable to conventional multifactorial risk factors rather than to laminopathy.

Parental mosaicism for *LMNA* variants has been previously reported across distinct laminopathy phenotypes. Wuyts et al. first described low-level maternal mosaicism (~10–15%) for the *LMNA* c.1824C>T mutation in a child with HGPS [18]. More recently, Wang et al. reported somatic and gonadal mosaicism in the mother of two siblings with *LMNA*-related cardiomyopathy, while Xie et al. demonstrated gonosomal mosaicism using ultra-deep sequencing and droplet digital PCR in a clinically unaffected carrier of the *LMNA* p.Arg541His variant [19,20]. These studies confirm that *LMNA* mosaicism can be clinically silent yet carries significant implications for recurrence risk and genetic counseling, emphasizing the importance of using highly sensitive detection methods capable of identifying low-level variants.

The clinical variability observed in laminopathies, including cardiomyopathies and progeroid syndromes, likely reflects the interplay of multiple factors—genetic, epigenetic, and environmental. Disruption of chromatin architecture and histone modification patterns has been shown to alter gene expression programs in *LMNA*-mutant cells, while external factors such as diet, lifestyle, and comorbidities further modulate disease expression. Cell-specific protein interactions with lamin A/C may also influence the spectrum and severity of tissue involvement. Such mechanisms may explain the variability observed in our familial case and among previously reported individuals carrying the same variant.

In conclusion, our findings expand the phenotypic and inheritance spectrum of *LMNA*-associated disorders by describing the first genetically confirmed familial case of nonsyndromic cardiac progeria due to the p.Asp300Asn variant with presumed gonadal mosaicism. This report underscores the need to consider *LMNA* variants in patients with early-onset cardiac disease and a positive family history, even in the absence of systemic features. Comprehensive genetic evaluation, sensitive detection of low-level mosaicism, and appropriate counseling are essential for accurate diagnosis, risk assessment, and timely clinical management aimed at reducing cardiovascular morbidity and mortality in affected families.

## 5. Conclusions

We report the first genetically confirmed familial case of nonsyndromic cardiac progeria associated with the LMNA, NM_170707.4:c.898G>A (p.Asp300Asn) variant, suggesting presumed gonadal mosaicism as a possible mechanism of inheritance. This case highlights the striking phenotypic variability linked to this variant, ranging from isolated cardiovascular disease to systemic progeroid features. Our findings underscore the importance of early genetic evaluation in patients with early-onset cardiac disease, particularly in the absence of conventional risk factors. Identification of pathogenic LMNA variants enables timely diagnosis, informed clinical management, and appropriate genetic counseling for affected individuals and their families.

## Figures and Tables

**Table 1:** The comparison of the clinical features between our case and previously published cases with NM_170707.4 (*LMNA*):c.898G>A (p.Asp300Asn).

Characteristics	Our Case	Mahdi et al., 2020 [4]	Marian et al., 2017 [9]	Motegi et al, 2014 [10]	Renard et al., 2009 [11]
**Sex**	f	f	f	m	m
**BMI (kg/m^2^)**	16.6	19	18.3	14.1	14.9
**Hypertension**	yes	yes	no	no	yes
**Family history**	father—(CABG) at 63 y sister—aortic stenosis, CAD, died at 43 y; brother—pulmonary embolism at 32y, died at 40 y	father—valvular heart disease, lean body habitus	N/D	sister—progeroid features, AVR, stroke at 47 y father and paternal relatives—stroke/MI, liver failure in midlife	father—Werner syndrome, MI at 52 y
**Facial/phenotypic features**	No phenotypic features	Lean body habitus with well-defined truncal and extremity musculature	No phenotypic features	High-pitched voice, pigmentation, sclerodactyly	Bird-like face, lipodystrophy, scleroderma-like skin
**Smoking**	no	no	no	no	no
**Diabetes mellitus**	no	no	no	no	no
**Serum cholesterol (mg/dL)**	64.62 (ref: 63–93.6)	Normal	130 (ref: <200)	High	Normal
**Serum Triglyceride (mg/dL)**	22.32 (ref: 9–31.5)	353, elevated (ref. N/D)	155 (ref: <150)	High	Normal
**Serum LDL (mg/dL)**	37.44 (ref: 1.44–72)	normal	75 (ref: <100)	High	Normal
**Serum HDL (mg/dL)**	17.1 (ref: 16.2–34.02)	31 reduced (ref. N/D)	24 (ref: 40–60)	N/D	N/D
**Other manifestations**		Hepatic steatosis Pancreatitis Sleep apnea Barrett’s esophagus			Progeroid features, basal squamous cell carcinomas

P—patient, y—year, f—female, m—male, CABG—coronary artery bypass grafting, MI—myocardial infarction, AVR—aortic valve replacement, N/D—no data.

## Data Availability

The datasets used and/or analyzed during this study are available from the corresponding author upon reasonable request.
